# Checks and balances? DNA replication and the cell cycle in *Plasmodium*

**DOI:** 10.1186/s13071-018-2800-1

**Published:** 2018-03-27

**Authors:** Holly Matthews, Craig W. Duffy, Catherine J. Merrick

**Affiliations:** Centre for Applied Entomology and Parasitology, Faculty of Natural Sciences, Keele University, Staffordshire, ST55BG, Keele, UK

**Keywords:** Malaria, *Plasmodium*, Cell cycle, Replication

## Abstract

It is over 100 years since the life-cycle of the malaria parasite *Plasmodium* was discovered, yet its intricacies remain incompletely understood - a knowledge gap that may prove crucial for our efforts to control the disease. Phenotypic screens have partially filled the void in the antimalarial drug market, but as compound libraries eventually become exhausted, new medicines will only come from directed drug development based on a better understanding of fundamental parasite biology. This review focusses on the unusual cell cycles of *Plasmodium*, which may present a rich source of novel drug targets as well as a topic of fundamental biological interest. *Plasmodium* does not grow by conventional binary fission, but rather by several syncytial modes of replication including schizogony and sporogony. Here, we collate what is known about the various cell cycle events and their regulators throughout the *Plasmodium* life-cycle, highlighting the differences between *Plasmodium*, model organisms and other apicomplexan parasites and identifying areas where further study is required. The possibility of DNA replication and the cell cycle as a drug target is also explored. Finally the use of existing tools, emerging technologies, their limitations and future directions to elucidate the peculiarities of the *Plasmodium* cell cycle are discussed.

## Background

**Fig. 1:**
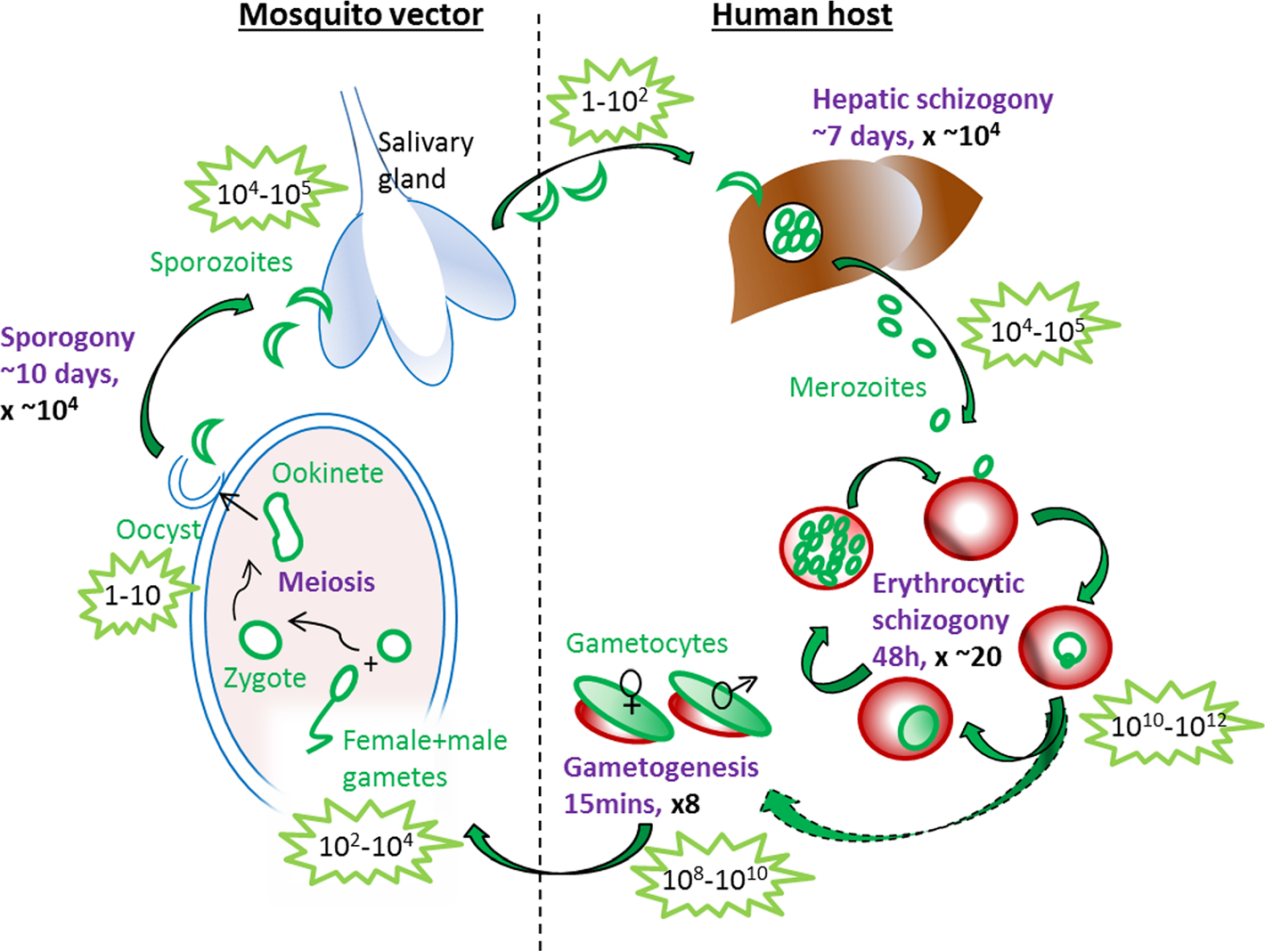
Schematic showing the life-cycle of *P. falciparum*. Each replicative stage of the life-cycle, together with the approximate fold-replication, is highlighted in purple. Approximate parasite numbers within each host at each stage are also shown to highlight the severe bottlenecks and massive expansions that occur throughout the life-cycle

The malaria parasite owes its success in part to its ability to ‘divide and conquer’ [1]. It pursues a complex, two-host life-cycle involving both mosquito and human hosts, in which each bottleneck is followed by a replication phase (Fig. 1). There are four periods of mitotic DNA synthesis and one period of meiosis during the course of the *Plasmodium* life-cycle [2]. The properties of cell division at these replication phases differ fundamentally from conventional models of eukaryotic cell division: rather than binary fission, the parasite opts primarily for schizogony whereby a multinucleate syncytium is formed, prior to budding and cytokinesis [3]. Equally intriguing is the remarkably rapid process of gamete formation, where male gametocytes undergo three rounds of DNA replication in a matter of minutes, producing eight male gametes [4, 5].

Such a complex life-cycle presumably requires sophisticated global and local regulators, involving refined checkpoint and DNA repair mechanisms [3], yet these are currently only poorly understood. Along with the unusual spatial and temporal dynamics of DNA replication, cell cycle regulators have been shown to be distinct from human counterparts [6]. Thus, replication in *Plasmodium* appears to be an excellent drug target: its mechanisms and regulators are distinct from those of the host organisms, the scale of reproductive output is directly crucial to pathogenicity, and it offers the possibility of interfering with the transmissibility of the parasite. Furthermore, the parasite possesses two organelles of bacterial origin, the apicoplast and mitochondrion, both of which carry their own genomes and may harbour distinct drug targets in the form of prokaryotic-type replication proteins (apicoplast replication was recently well-reviewed [7], so this article focusses only on nuclear replication). Finally, in the current era of artemisinin resistance, which appears to involve parasite ‘dormancy’, understanding cell cycle arrest and checkpoints is of utmost importance.

## The *Plasmodium* cell cycle

**Fig. 2:**
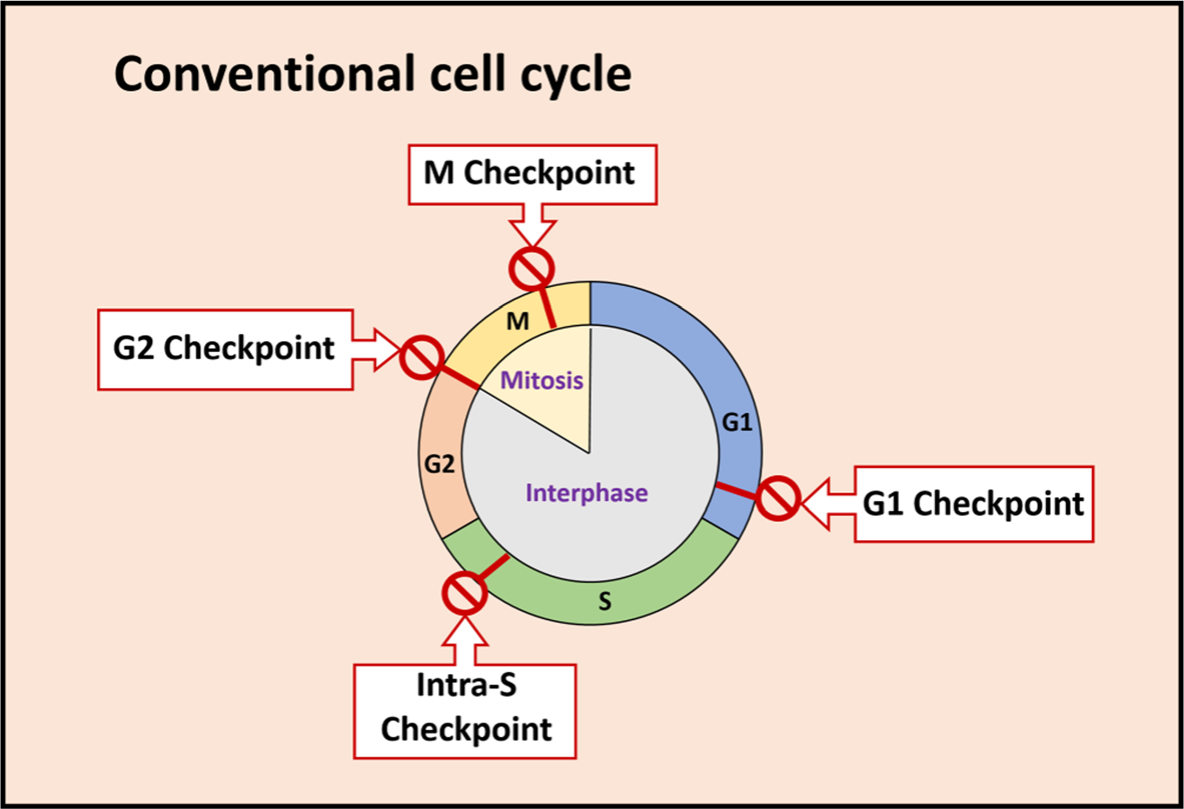
Schematic of the conventional eukaryotic cell cycle, highlighting the points at which cycle checkpoints operate

The standard eukaryotic cell cycle follows a clearly defined series of stages during which the cell grows (interphase), replicates its chromosomes (S phase) and divides (M phase), with S phase often being flanked by two gap phases called G1 and G2 (Fig. 2). The process is tightly regulated by an extensive regulatory network that ensures the cell is ready to progress onwards through the cycle after each phase [8, 9]. Apicomplexans, including *Plasmodium* spp., deviate significantly from this classical model, allowing each cell to produce dozens or hundreds of new daughter cells during a single replicative cycle. At the heart of these adaptations is schizogony: a syncytial and yet asynchronous form of replication and cell division, which occurs in the mammalian host during hepatic and erythrocytic infections, while a largely analogous process called sporogony occurs in the mosquito vector during oocyst formation (Fig. 1).

For the most virulent human malaria parasite, *P. falciparum,* the parasites in the erythrocytic cycle, when grown *in vitro*, produce an average of 16 daughter merozoites from a single infected erythrocyte. Of these, about two-thirds can successfully invade new erythrocytes, resulting in growth rates of up to 11-fold per 48-hour cycle [10, 11]. Infected hepatocytes and oocysts have been less closely studied, but each of these can produce many thousands of daughter cells during a single replicative cycle, taking ~ 7–10 days. Finally, the extremely rapid replicative process of male gametogenesis takes just 10–15 minutes (Table 1). Thus, there are huge variations of both speed and magnitude between the various replicative stages of the *Plasmodium* life-cycle.

**Table 1:** Summary of replicative stages in the *P. falciparum* life-cycle

Replicative stage	Host	Time period	Daughter cells	Rounds of replication	Time available per genome replication	Key references
Hepatic schizogony	Human (liver)	~ 7 days	> 10,000	~ 14	~ 12 h	[108, 109]
Erythrocytic schizogony	Human (bloodstream)	48 h	~ 16–20	4–5	~ 4 h	[13]
Gametogenesis	Mosquito	15 min	8	3	< 4 min	[4, 110]
Sporogony	Mosquito	~ 10 days	~ 10,000	~ 14	~ 17 h	[111, 112]

## Cell-cycle mechanics at the cellular level

The *Plasmodium* cell cycle is best characterised during erythrocytic schizogony, with the start of each cycle being associated with the invasion of a new erythrocyte by a merozoite. During the first 24 hours post-invasion (hpi), ring and early-trophozoite parasites possesses a single haploid nucleus in interphase or G1. The centriolar plaque (CP), which is functionally equivalent to the centrosomes in higher eukaryotes, begins to assemble and duplicate 20–24 hpi, marking the shift from growth to replication. Replication of the chromosomes initiates at estimated times of 29 hpi [12] or 24–26 hpi [13], overlapping with the semi-conservative duplication of the CP. It was initially proposed that several rounds of continual chromosome replication would then occur, followed by a single co-ordinated mass chromosome segregation, nuclear division and formation of daughter merozoites [14]; however, an alternative model entails repeated cycles of replication, segregation and nuclear envelope division, based upon the observed segregation patterns of the CP [13]. In this model the characteristic asynchronicity of schizogony correlates with the inheritance of the CP, with nuclei that inherit the larger mother CP being ready to divide sooner than those nuclei with the daughter CP.

It is not known what signal induces a schizont to stop its asynchronous nuclear divisions and undergo a final - and apparently synchronous [13] - division, followed by budding into membrane-bound daughter cells. Space or nutrients within the host erythrocyte may become limiting, or the timed production of a global cell cycle regulator may be hard-wired into *Plasmodium*’s transcriptional cascade [15]. The latter model could potentially enforce synchronicity in the final nuclear division, but it would presumably run the risk of ‘catching’ existing nuclei at different stages of S phase. Streipen and co-workers, working largely in the related apicomplexan parasite *Toxoplasma gondii*, have proposed that ‘local’ *versus* ‘global’ control is centrally coordinated by the CPs [3, 16], while a remnant flagellum is the organising principle for daughter cell budding [17]. This may also be true for *Plasmodium*, although schizogony is numerically more complex than endodyogeny in *T. gondii* and the challenges of its coordination may in fact be more analogous to the replication of hyphal yeasts [18]. Overall, significant gaps remain in our understanding of the cell biology underlying schizogony, and even less is known about the other phases of *Plasmodium* replication (Fig. 1), for which erythrocytic schizogony may yet prove an inadequate model.

## Mechanics of DNA replication at the molecular level

Many early-diverging eukaryotes, such as *Trypanosoma* and *Oxytricha*, organise their genomes in extremely unusual ways [19, 20]; by comparison, the basic genome structure of *Plasmodium* is quite conventional. There are 14 linear chromosomes with telomeres and centromeres, plus two small organellar genomes in the mitochondrion and apicoplast [21]. This conventional genome is replicated, however, within the framework of fundamentally unusual cell biology. Although much remains to be studied, some of the proteins and parameters involved in this process have now begun to be elucidated.

*Plasmodium* encodes the basic replicative machinery that is found in all eukaryotes, including DNA polymerases [21, 22], proliferating cell nuclear antigen (PCNA) [23, 24] and minichromosome maintenance proteins (MCMs) [25, 26]. Components of the Origin Recognition Complex (ORC) are also present although only two ORC components have been characterised thus far in *P. falciparum*, *Pf*ORC1 and *Pf*ORC5 [14, 27], while a putative ORC2 has been shown to be upregulated during gametocyte activation in *P. berghei* [28]. The remaining members of the complex are either absent or lack sufficient homology with characterised members to allow clear identification, although in *P. berghei* putative homologues of ORC4 and ORC3 have recently been proposed [28]. The ORC-associated protein Cdc6 also lacks a clear homologue in *P. falciparum* but a candidate has been proposed in *P. berghei* [28]. Cdc6 is required for the recruitment of Cdt1 and the loading of MCM proteins (to date only a putative Cdt1-like gene has been identified in *Plasmodium*). Interestingly, *Pf*ORC1 shares a homology region with Cdc6 at the C-terminus and it may, therefore, be regulated in a Cdc6-like manner. In *S. cerevisiae* and humans, the onset of Sphase results in the recruitment of two further components to activate the MCM helicase: Cdc45 and the GINS complex (consisting of Sld5, Psf1, Psf2 and Psf3). Sequence analysis has failed to identify a clear homologue of Cdc45 in *Plasmodium*, while the members of the GINS complex have been putatively identified, based on low sequence homology, but it remains to be determined whether they are functional.

**Fig. 3:**
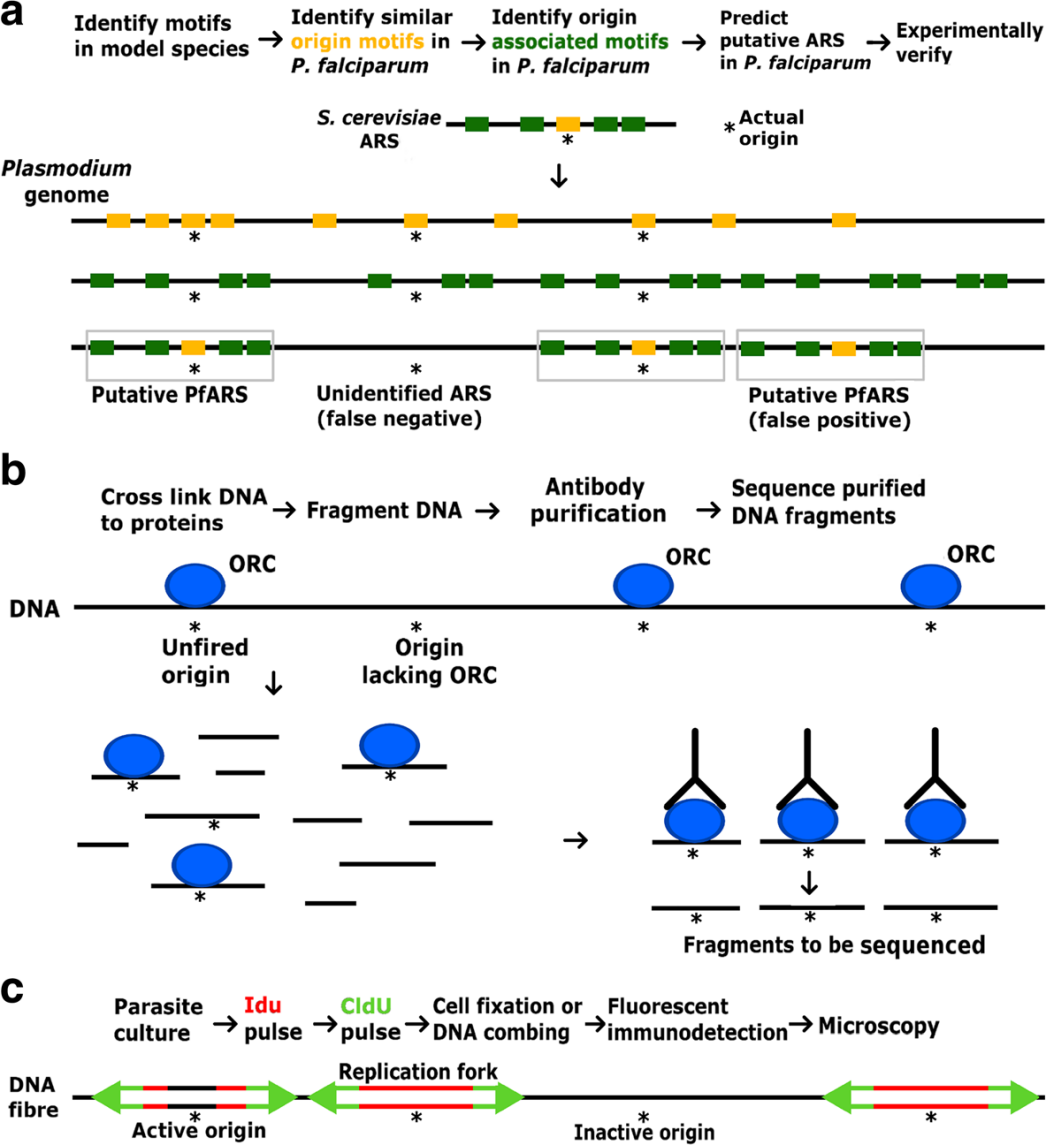
Development of techniques to examine the replication of the *Plasmodium* genome. **a** Bioinformatic analysis of conserved sequences at and surrounding replication origins in the *S. cerevisiae* genome has led to identification of common motifs for searching the *Plasmodium* genome [30]. Origins in *S. cerevisiae* consist of compact autonomously replicating sequences (ARS) with ‘A domain’ motifs (orange) and surrounding ‘B domains’ (green). The *Plasmodium* genome has a high concentration of individual A and B domains (~ every 2500 bp) but a much lower concentration when the requirement for closely associated domains is imposed (grey boxes). Bioinformatics approaches can only identify putative origin sites and may fail to identify true origins (*) or identify sequences which are incapable of functioning as origins. **b** Chromatin immuno-precipitation (ChIP) of the proteins required before and during replication, such as members of the Origin Recognition Complex (ORC), allows experimental characterisation of origin sequences [30]. Following reversible DNA-protein cross linking, the genome is fragmented and the proteins of interest are purified along with the associated DNA fragments, which are then sequenced. This may include origins that would never be activated, and may miss those where the protein complex has dissociated from the chromosome. **c** Synthetic nucleoside labelling and DNA combing techniques allow the labelling and fluorescent immunodetection of *de novo* DNA synthesis [31]. Parasites expressing viral thymidine kinase can incorporate the synthetic nucleosides IdU (red) and CldU (green) which can be visualised in individual nuclei or on combed DNA fibres, allowing the calculation of inter-origin distances and replication rates. The synthetic nucleosides will only be incorporated around active origins (*) while inactive origins will remain unlabelled and therefore undetected

ORC binds DNA and facilitates the initiation of replication. It recognises a conserved consensus sequence in the yeast *Saccharomyces cerevisiae*, but in other eukaryotes there is no consensus sequence and the preferred composition of DNA bound by ORC varies from organism to organism [29]. Putative ORC-binding sequences in *P. falciparum* have recently been identified *in silico*, based on the AT-rich ORC-binding site of *S. cerevisiae* (Fig. 3a): they are spaced only ~ 2500 bp apart in this highly AT-rich genome and although selected sites do show significant enrichment of ORC1 in ChIP assays [30] (Fig. 3b), only a small proportion of these sites appear to be used for intra-erythrocytic replication. DNA ‘combing’ of labelled DNA fibres was recently used to investigate the actual distribution of active replication forks during erythrocytic schizogony (Fig. 3c) and this revealed a mean distance of 65 kb between individual origins, with replication forks moving at a mean rate of 1.19 kb/min [31]. The rate of replication was not constant, but decreased as the cells neared completion of schizogony, coinciding with a reduction in the mean distance between individual origins. Interestingly, this is the opposite of the pattern seen in human cells, where replication speeds up and origins become more widely spaced as S phase proceeds. In a schizont, which is replicating not 2-fold but ~ 16-fold, the availability of nucleotides, the physical space available or the increasing compaction of chromatin may all limit the speed of replication as S phase advances [31].

During erythrocytic schizogony the first replication (and possible nuclear division) requires 4–6 hours [13]. This, however, is not the maximal rate of replication for the parasite. After ingestion by a mosquito, male gametocytes undergo a 3-fold replication of their genome in less than 15 minutes, resulting in the production of 8 motile microgametes with 1N genome content [4, 5]. (Female gametocytes also mature and exit their host cells upon entering a mosquito but no replication or cell division occurs.) In the model rodent malaria species *P. berghei*, in which much of the work on gametocytes has been conducted, the first round of replication is usually completed within a minute of gametocyte activation [32]. Such extreme speed is unprecedented in eukaryotic gametogenesis, and may reflect strong pressure to complete the sexual cycle and exit the midgut before the parasite cells are digested along with the blood meal. It has yet to be determined whether the replication rate, the number of simultaneously active origins or both are significantly increased during gametocyte replication but the question of replication fidelity clearly becomes particularly pressing at this unique point in the parasite’s life-cycle. If the replication speed remains the same as it is in erythrocytic schizogony, then almost all of the suggested ORC-binding sites [30] must be used as origins simultaneously. Precedents do exist for such extremely flexible origin usage: in the earliest replications of *Xenopus* embryos, origins occur every 5–15 kb, spacing out only after the mid-blastula transition [33]. *Plasmodium* genome replication may be under similarly flexible control, although nothing is yet known about how this might be differentially enforced in gametogenesis, sporogony, hepatic and erythrocytic schizogony.

## Regulation of the *Plasmodium* cell cycle

As described above, *Plasmodium* undergoes multiple unconventional cell cycles, in a variety of host cell types and for varying durations. The intricacy and temporal/spatial accuracy with which these cell cycles are governed requires global and local regulators that must be fine-tuned and potentially equally unconventional. Although the genomic revolution for *Plasmodium* has permitted some investigation of these regulators, our understanding at present is patchy and incomplete.

### Cyclins and CDKs

In eukaryotic cells, cell cycle progression is governed by cyclins and cyclin-dependent protein kinases (CDKs), along with other proteins such the anaphase promoting complex (APC), which promotes waves of cyclin degradation. The interplay between these regulatory and catalytic components and their timely upregulation, inhibition and degradation prompts sequential progression through G1, S, G2 and M phases [34] (Fig. 2).

**Fig. 4:**
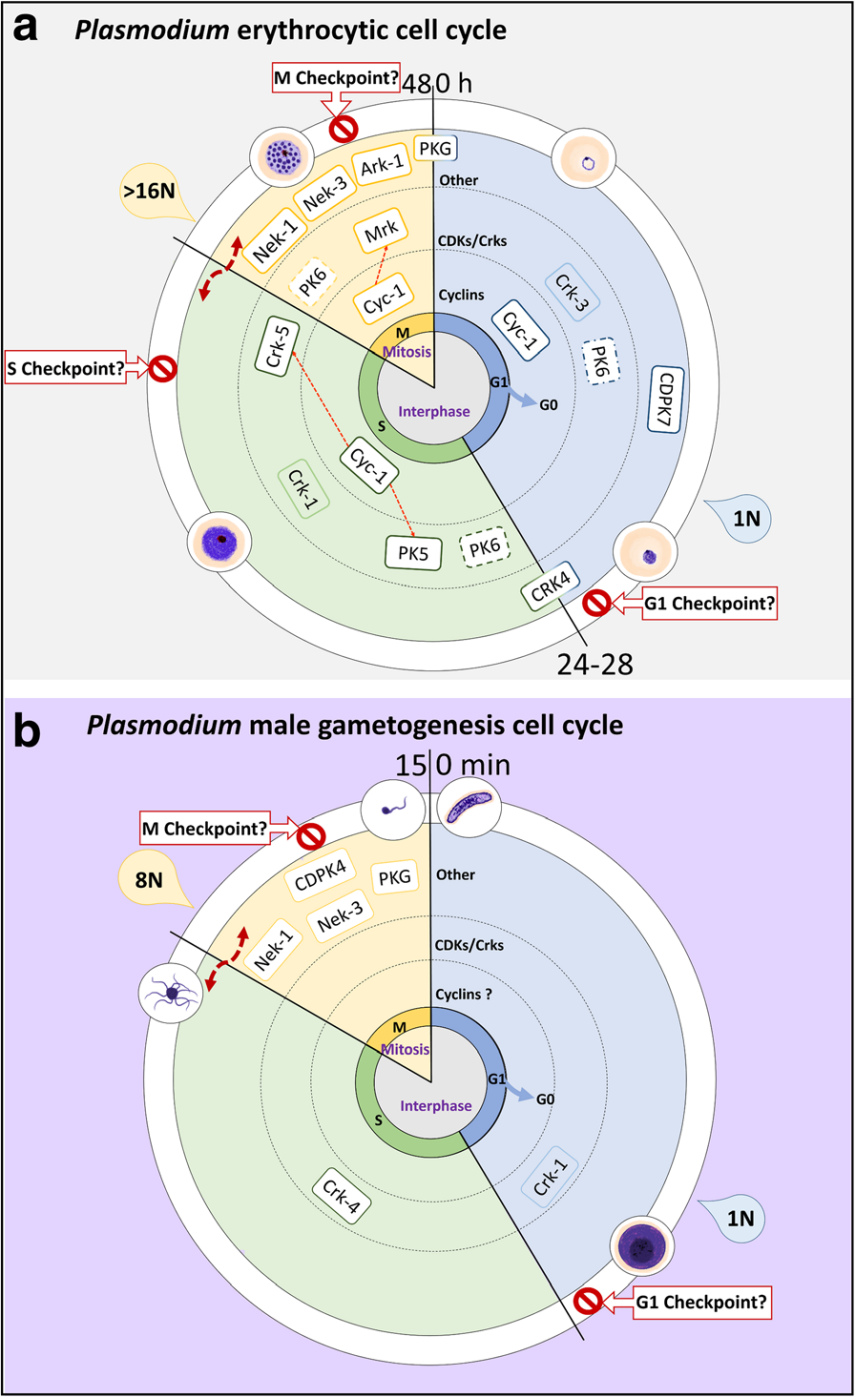
Illustration of cell cycle phases in *Plasmodium* erythrocytic schizogony (**a**) and *Plasmodium* male gametogenesis (**b**). The predicted involvement of cyclins, CDKs and other kinases is shown at each phase. Placement of such components is only loosely chronological since most details are unknown. CDKs/Crks with a dashed outline indicate cyclin independence. Crks or CDKs predicted to be involved in transcriptional regulation are transparent (without a white background). Interactions identified *in vitro* between cyclins and CDKs are indicated by a dashed orange arrow. Table 2 identifies all sources used to construct the figure. The cell cycles at sporogony and hepatic schizogony are omitted due to the lack of information about these stages

The peculiarities of *Plasmodium* schizogony begin with the lack of a G2 phase as the syncytial nuclei appear to alternate asynchronously between S and M phases prior to the orchestrated event of cytokinesis [35] (Fig. 4a). This raises questions about whether control of replicative cycles through diffusible cytoplasmic factors is feasible [2, 12]. Although such factors may exist, the Apicomplexa have a very unusual repertoire of cyclins, CDKs and CDK-related kinases (CRKs): in total, three cyclins, Cyc1, Cyc3 and Cyc4 [6], and seven CDK or CDK-related kinases, PK5, PK6, Mrk1, Crk-1, Crk-3, Crk-5 [36] and Crk-4 [12], have been identified in *Plasmodium* (Table 2).

**Table 2:** Regulators and their action in *Plasmodium* cell cycles

Regulator		Stage of life-cycle^a^	Cell cycle action	Gene ID	Reference
Cyclin	Cyc1	Erythrocytic stages (*Pf)* (peak expression in trophozoite)	Binds PfPK5 (*in vitro*) and PfMrk1 (*in vivo*). Role in segmentation	PF3D7_1463700; PBANKA_1233200	[44, 46, 113]
	Cyc3	Oocyst formation (*Pb*)	Binds PfPK5 (*in vitro*)	PF3D7_0518400 (putative); PBANKA_1233200	[6]
	Cyc4	Unknown	Activates PfCrk-5 *in vitro*	PF3D7_1304700; PBANKA_1403200 (putative)	[43]
CDKs	PK5	Erythrocytic schizogony (*Pf*)	DNA synthesis	PF3D7_1356900; PBANKA_1133200	[40, 45]
	Mrk	Erythrocytic schizogony (*Pf*) (mRNA, however, more abundant in gametocytes than in asexuals)	Cytokinesis in erythrocytic schizogony (*Pf*). Transcriptional regulator?	PF3D7_1014400; PBANKA_1212800	[38, 44, 46]
	PK6	Erythrocytic schizogony (trophozoite)	Onset of S phase (cyclin independent)	PF3D7_1337100; PBANKA_1350900	[47]
Crks	Crk-1	Gametocytes (*Pf*) Erythrocytic schizogony (*Pb*)	Transcriptional regulator	PF3D7_0417800; PBANKA_0719900	[114, 115]
	Crk-3	Erythrocytic schizogony (*Pf*)	Transcriptional regulator	PF3D7_0415300; PBANKA_0717300	[42]
	Crk-5	Erythrocytic schizogony (*Pf*)	Proliferation - number of merozoites. Activated by Cyc 1 and 4 *in vitro.* Cyclin independent?	PF3D7_0615500; PBANKA_1230200	[43]
Other Kinases	CDPK4	Gametogenesis (*Pb*)	Mitotic spindle assembly	PF3D7_0717500; PBANKA_0615200	[28]
	CDPK7	Erythrocytic schizogony (*Pf*)	Unknown	PF3D7_1123100; PBANKA_0925200	[53]
	Crk-4	Erythrocytic schizogony (trophozoite/schizont) (*Pf*)	S phase	PF3D7_0317200; PBANKA_0808000 (putative)	[12]
	Nek-1	Erthrocytic schizogony/Gametogenesis (*Pb*)	Mitosis	PF3D7_1228300; PBANKA_1443000 (putative)	[116]
	Nek-2	Zygote (*Pb*)	Meiosis	PF3D7_0525900; PBANKA_1240700	[49, 50]
	Nek-3	Erythrocytic schizogony/Gametogenesis (*Pb*)	Unknown	PF3D7_1201600; PBANKA_0600600 (putative)	[117]
	Nek-4	Zygote (*Pb*)	Meiosis	PF3D7_0719200; PBANKA_0616700	[49, 50]
	Ark1	Erythrocytic schizogony/Gametogenesis (*Pb*)	Mitotic spindle formation/ Cytokinesis	PF3D7_0605300; PBANKA_0104100	[52]
	Ark2	Erythrocytic schizogony/Gametogenesis (*Pb*)	Mitotic spindle formation/ Cytokinesis	PF3D7_0309200; PBANKA_0407400	[52]
	Ark3	Erythrocytic schizogony/Gametogenesis (*Pb*)	Mitotic spindle formation/ Cytokinesis	PF3D7_1356800; PBANKA_1133100	[52]

^a^Information has been compiled from studies completed with either *Plasmodium falciparum* (*Pf*) or *Plasmodium berghei* (*Pb*) as indicated

None of the cyclins are homologs of canonical cell-cycle cyclins (e.g. mammalian Cyc D, E and A) and they tend to be ubiquitously expressed at many stages and not to ‘cycle’ as seen in mammalian cells [6, 37]. Amongst the CDKs, PK5 is the only enzyme that clusters with mammalian cell-cycle CDKs; of the others, Mrk1 is the putative functional homologue of CDK7, which is both a CDK-activating kinase and a component of the general transcription factor TFIIH [38–40], Crk1 and Crk3 are homologous to transcriptional CDKs, and PK6 and Crk5 are ‘atypical’ CDKs specific to Apicomplexa [41–43].

Although PK5 is the putative homologue of mammalian CDK1, *Plasmodium* encodes no cognate cyclins for such an enzyme and the activator for PK5 remains unknown: *in vitro*, it is unusually promiscuous and can be activated by all three *Plasmodium* cyclins as well as mammalian p25, cyclin H and RINGO [37, 44, 45]. The partnership with Cyc1 is questionable because recent immunoprecipitation studies failed to identify it [46]. Nevertheless, PK5 has been shown to be involved in ORC1 phosphorylation, implicating it in DNA replication in erythrocytic stages [45]. Mrk1 is not apparently a CDK-activating kinase in *Plasmodium* and although it can interact *in vitro* with the replication factor complex (PfRFC-5) and PfMCM6 [40], it actually appears to be crucial for cytokinesis rather than replication, as indeed is Cyc1 [46], with Mrk1 acting in a complex with Cyc1 and MAT1 [46]. Crk5 can be activated *in vitro* by Cyc1 and Cyc4 but its *in vivo* partner is again unknown; it is involved in, but not essential for, erythrocytic schizogony because its absence results in viable parasites with fewer merozoites per schizont [43]. PK6 is proposed to be involved in the onset of S phase in erythrocytic stages but *in vivo* characterisation is lacking, and recombinant PK6 is cyclin-independent *in vitro* [47]. The remaining CDKs, Crk-1 and Crk-3, are predicted to have roles in transcriptional regulation and thus in cell growth and proliferation [42, 48]. Overall, it is clear that *Plasmodium* cell cycles are not regulated by conventional waves of cyclin/CDK activity, although schizogony and sporogony do require Cyc1 and Cyc3, respectively [6, 46], and several CDKs are evidently involved.

### Non-cyclin/CDK cell cycle regulators

A host of cell cycle regulators beside the cyclins/CDKs have been identified in *Plasmodium* (Fig. 4). Some of these are homologs of conventional eukaryotic regulators, including the NIMA kinases (Nek1-4) and the Aurora kinases (Ark1-3): Nek1 plays an essential roles in mitosis [43] and Nek2-4, in gametogenesis/meiosis [49–51], while the Arks are involved in mitotic spindle formation and cytokinesis [52]. Another - perhaps more interesting - group of regulators are specific to the unusual cell cycle modes of apicomplexans and there is considerable interest in the plant-like calcium-dependent protein kinases (CDPKs) as possible parasite-specific drug targets, with CDPK4 playing multiple roles in male gametogenesis [28] and CDPK7, in erythrocytic schizogony [53]. Another *Plasmodium*-specific kinase, PfCRK4, was recently identified as essential for DNA replication in erythrocytic schizogony, although the pathway in which it acts remains to be elucidated [12]. The regulation of *Plasmodium*’s atypical cell cycles could clearly be rich source of novel drug targets, but much work is still required to piece together the molecular signalling pathways involved.

## Cell cycle checkpoints in *Plasmodium*

In addition to the cyclin-CDK regulatory network, there are also defined checkpoints in yeast and mammalian systems that control cell cycle advancement. These serve as quality control for cell growth (G1 checkpoint), successful DNA replication or DNA damage (S and G2 checkpoints) and chromosome attachment to the spindle (M checkpoint) [54]. Checkpoints are particularly important for avoiding re-replication and preventing the propagation of incompletely replicated or damaged daughter genomes.

The existence of cell cycle checkpoints in *Plasmodium* remains, in general, uncertain, and genes encoding key checkpoint proteins such as Rb, p53, ATM and ATR have not been identified. There is, however, some evidence of a G1 checkpoint in the related parasite *T. gondii* [55], and blood-stage *Plasmodium* parasites can induce a comparable, reversible rest state/dormancy at the prereplicative ring stage in response to nutrient starvation [56] and drug pressure [57–59]. What induces these states in the absence of a conventional G1 checkpoint pathway is unclear.

DNA repair machinery is largely conserved in the parasite genome, as described below, and parasites respond to DNA damage by upregulating repair machinery and altering chromosome structure [60]. However, there is no apparent G2, offering no opportunity for a G2 checkpoint [13, 35] and the feasibility of intra-S and M checkpoints is challenged by the striking variation in the speed of genome replication at different life-cycle stages, particularly the unprecedented rate in male gametocytes [61], sharply contrasting with a more conventional rate during erythrocytic schizogony [13, 31]. Checkpoint regulation may be temporally possible during erythrocytic schizogony - and perhaps also oocyst sporogony and hepatic schizogony - but not gametogenesis. Spatially, schizogony also poses challenges to checkpoint control. Although chromosomes do appear to align with the hemispindle, which is anchored to a CP, they remain uncondensed: it is thought that the centromeres remain constantly attached to CPs and that this may help to separate the uncondensed chromosomes accurately [3]. Finally, the syncytial nature of *Plasmodium* replication raises questions about diffusible checkpoint factors and the how the replication of individual genomes could be stalled within a shared cytoplasm [2]. Nevertheless, the observation that schizonts produce very varied numbers of merozoites while the erythrocytic cycle is always ~ 48 hours [62] does imply that intra-S or M checkpoints may delay some nuclei, and that the time span of schizogony is not imposed by the number of merozoites to be produced.

Variations in cell cycle speed also raise questions about replicative fidelity and tolerance of karyotypic variation. Indeed, this parasite’s relentless ability to develop resistance against all antimalarials is undoubtedly linked to genomic plasticity, but it remains debatable whether plasticity is inherent in the *P. falciparum* genome, or is actually promoted by unusually relaxed cell-cycle control. Under drug pressure, *P. falciparum* erythrocytic stages can initiate AT-track mediated, random duplication of segments of the genome, followed by the establishment of pseudopolyploids around a high priority locus. This permits fine-tuning of amplicon numbers, relevant to drug pressure, while avoiding genome damage and any deleterious mutations in off-target loci [63]. In fact, 20 years ago the concept of ‘hypermutator’ strains specific to certain global regions was proposed to explain the fact that drug-resistant parasites regularly arise in Southeast Asia [64], but this has proved difficult to evidence, or to link it to any molecular defect in DNA repair or checkpoints [65, 66]. Two recent studies have suggested that the mitotic mutation rate does not vary between *P. falciparum* strains and is not increased by drug pressure; rather, beneficial resistance mutants are simply selected under drug pressure [67, 68]. In this regard, *P. falciparum*’s active maintenance of a highly AT-biased genome (81% A/T), with high insertion/deletion rates and an overrepresentation of homopolymeric A/T tracts [69], may be the key to rapid adaptive evolution [70].

The extremely fast replication of male gametes raises a particular conundrum in terms of checkpoints: does this phase require especially stringent regulation, or conversely, more relaxed control to favour speed over fidelity? The observation that some male gametes are produced with apparently partial or absent nuclei (unpublished observations) and the fact that male-expressed genes display fast rates of evolution [71] may suggest the latter. Proteins involved in DNA replication and mitosis are simultaneously phosphorylated within the first 20 seconds of gametocyte activation, contrary to the traditional view of sequential progression through the cell cycle, and this may facilitate the rapidity of gametogenesis [32]. Indeed, the relatively limited repertoire of cell cycle kinases in *Plasmodium* may also imply that some have dual functions: CDPK4 has been implicated in assembly of the pre-replicative complex, mitotic spindle formation, cytokinesis and axoneme motility [28, 32, 72]. Extending this concept, highly divergent kinases may also operate ‘incognito’ as checkpoint kinases in *Plasmodium.*

Regardless of cell cycle speed, the parasite is clearly able to promote genomic diversity during mitosis, as well as more conventionally at meiosis. It seems unlikely that the intricacy and precision of the *Plasmodium* cell cycles would proceed unchecked, but evidence is currently lacking for clearly defined checkpoints and there may be great flexibility in which checkpoints are enforced during different types of replication.

## DNA repair in *Plasmodium*

DNA damage can originate from a range of sources, the most common in *Plasmodium* being reactive oxygen species generated by metabolism, free radicals, which are often produced after uptake of antimalarial drugs such as chloroquine or artemisinin, and errors made during DNA replication. Damage may affect individual bases or may lead to the generation of potentially deadly double strand breaks (DSBs). The mutational spectrum observed in *P. falciparum* is highly unusual: the SNP mutation rate is 2.45 × 10^-10^ mutations per base pair per life-cycle but this is dwarfed by a 10-fold higher indel mutation rate, driven by low complexity AT-rich sequences and a significant G:C -> A:T transition bias [70]. This can promote the formation of pseudopolyploid loci, as described above, but the core genome nevertheless remains intact, due to the presence of an effective DNA repair system including most - although notably not all - of the pathways commonly found in model eukaryotes.

Damage to individual bases is resolved by the excision repair pathways that include nucleotide excision repair (NER), base excision repair (BER) and mismatch repair (MMR). Orthologs of the majority of genes involved in the NER pathway have been identified bioinformatically, with the exception of p62 and XPC [73]. Similarly, the majority of the MMR pathway is present but there are notable differences from other eukaryotes, with RecJ exonucleases appearing to be absent while a UvrD helicase homolog, found in *E. coli* but absent in humans, is present [74, 75]. *Plasmodium falciparum* lacks homologs for short-patch BER but a long-path BER pathway is present [76].

The majority of eukaryotes rely upon two major pathways for the repair of double-strand breaks, homologous recombination (HR) and non-homologous end joining (NHEJ). The *Plasmodium* genome encodes a functional HR pathway but the core genes of the NHEJ pathway appear to be absent across the genus [21, 77], supported by the inability to detect NHEJ products *in vitro* after the experimental generation of DSBs [78, 79]. Accordingly, HR is the prime source of DSB repair in the parasite [79–81] (by contrast, *T. gondii* possesses a functional NHEJ pathway, indicating loss of this pathway in *Plasmodium* [82, 83]). HR, indeed, appears to be essential for the completion of the parasite life-cycle because the knockout of a zinc finger protein, *Pb*Zfp, in *P. berghei* leads to a loss of transmission competence in mosquitoes due to a failure to recruit the topisomerase-like enzyme Spo11 to recombination hotspots [80]. During all haploid growth phases the parasite must therefore rely upon alternative end joining pathways such as microhomology-mediated end joining (MMEJ) to repair DSBs within the core genome, because no repair template exists to allow HR [84]. Bioinformatic comparisons with the *S. cerevisiae* and human genomes have identified *Plasmodium* orthologs of the MMEJ components, but studies of irradiated parasites suggest that the process is inefficient or used infrequently, and that parasites with a 1N genome content are unable to repair damage as efficiently as trophozoites with genome content of 2N or more [85–87].

Notably, this restriction does not apply to multigene families, such as the *var* family of key virulence genes in *P. falciparum*. There are ~ 60 hypervariable *var* gene homologs in every parasite genome so these can be repaired *via* HR even in haploid parasites, using homologs within the same family. This leads to important diversification of these gene families during mitotic growth [68] (as well as during meiosis), generating new antigenic variants that can facilitate immune evasion during chronic human infections. *Var* gene recombination does not require substantial stretches of high sequence homology and the genes do not necessarily recombine with their closest homologues [68]; the physical clustering of *var* genes at the nuclear periphery may favour sequence pairing even in the absence of extensive homology.

## DNA replication in *Plasmodium* as a potential drug target

Historically, DNA replication has been an excellent drug target in malaria parasites, as demonstrated by Fansidar: an anti-folate drug combination which proved vital after the emergence of chloroquine resistance in the late 1950s [88]. Fansidar is a synergistic combination of two drugs that block the pathway to production of reduced folate cofactors (essential for nucleotide production and DNA synthesis), but resistance to the combination arose fairly rapidly [89, 90]. However, directly targeting the regulatory machinery of the parasite, such as cell-cycle checkpoint control, or eliciting DNA damage as a route to parasite killing, may provide a greater hurdle to resistance development. Indeed, DNA damage, together with protein damage, is thought to be a mode of action for the frontline antimalarial drug artemisinin, mediated through free radicals [91]. *Plasmodium falciparum* responds to artemisinin by inducing dormancy at G0/G1, downregulating DNA synthesis-related cyclins/CDKs, upregulating putative ‘negative-regulatory’ cyclins/CDK’s [35] and upregulating DNA repair machinery - specifically, PfRad51, PfRad54, PfRPAIL and PfRPAIS [60, 92]. Artemisinin has also been shown to induce G0/G1 arrest in *Leishmania donovani* promastigotes [93] and in multiple cancer cell lines [35]. For *Plasmodium*, understanding the cell cycle arrest phenotype takes on new urgency because it is considered a key mechanism of artemisinin resistance [94]. This resistance is not yet fully understood in molecular terms, but it correlates with mutations in the Kelch-13 protein, which in turn correlate with elevated levels of the phosphoinositide-3-kinase enzyme PfPI3K [95]. PfPI3K, a lipid kinase, is distantly related to protein kinases that are key checkpoint proteins in most eukaryotes but are missing in *Plasmodium* - an intriguing similarity that is currently under investigation in our laboratory.

The ability of resistant parasites to survive in a dormant state and recrudesce weeks later may be exacerbated by the recent finding that erythrocytic schizogony is remarkably flexible in resistant parasites. Resistant clones from Southeast Asia have a prolonged ring stage and considerably shortened trophozoite stage, presumably reducing drug exposure to the more vulnerable trophozoite [57]. This phenotype was stable without artemisinin drug pressure, which is particularly worrying because it could reduce the available window for antimalarials that target the trophozoite stage and that may be used as partner drugs in artemisinin combination therapies, or as alternative drugs if such therapies fail.

Artemisinin resistance first arose at the Thai/Cambodian border, the historic epicentre of drug resistance development, and Cambodian isolates have been shown to have a mild mutator phenotype and mutations in a number of DNA repair genes, including members of the mismatch repair pathway Mlh1, Pms1 and Exo1 [66]. (This is in addition to mutations in the Kelch-13 propeller domain [96], which serves as a molecular marker for resistance). In bacterial systems, mild mutators acquire mutations at a lower rate than hypermutator lines but remain able to purge deleterious mutations efficiently from the genome and thus they can outcompete hypermutators in the long term [97–100]. A fine balance is clearly required between the need for accuracy and adaptability, and this balance has probably shifted in *P. falciparum* in response to continual pressure from antimalarial drug programmes. By analogy with cancer cells - which frequently have deficient checkpoint and DNA repair pathways - artemisinin resistant parasites may be on a knife edge between efficient growth and the potential disaster of an under-regulated cell cycle, so it may be possible to target them with drugs that exacerbate their defects. Proteasome inhibitors can synergise with artemisinin by exacerbating the effects of protein damage [101], so perhaps a similar strategy to inhibit a crucial checkpoint or DNA repair pathway could exacerbate the effects of artemisinin-induced DNA damage.

A better understanding of cell cycle regulation, checkpoints and repair mechanisms in *P. falciparum* is needed to aid the discovery of compounds that specifically inhibit checkpoints or DNA repair. There may also be scope for drugs that target the cell cycle itself, since components such as the cyc-CDK machinery are distinct from human counterparts. Indeed, like many *Plasmodium* proteins, some conserved replication proteins also have distinct N-terminal extensions or inserted domains that could serve as highly specific drug targets. Such discoveries could not only circumvent artemisinin resistance but could also identify a much-needed novel class of antimalarial agents. Recently, the CDK inhibitors olomoucine (PfCRK1, PfPK5 and PfPK6 inhibitor), roscovitine (PfPK5 and PfPK6 inhibitor) and WR636638 (chalcone inhibitor of PfMRK) were shown to have differential effects on artemisinin induced dormancy [35]. The DNA synthesis inhibitor aphidicolin and PfPK5 inhibitor flavopiridol also decrease DNA replication in malaria parasites [102]. Overall, the very fact that artemisinin is effective validates DNA damage as a drug target, but the looming catastrophe of widespread artemisinin resistance urgently demands a better understanding of the resistance mechanism, its relationship with DNA repair machinery, and with the control of the *Plasmodium* cycle cell as a whole.

## State-of-the-art focus: Experimental techniques to study the *Plasmodium* cell cycle

Investigation of the *Plasmodium* cell cycle has been hampered a lack of transferable techniques from other well-studied systems. Erythrocytic schizogony has been most thoroughly investigated because the blood stages of *P. falciparum* are relatively easy to culture. However, during schizogony the small size of nuclei (diameter > 1 μm) limits the resolution of immunofluorescence microscopy and the multiple asynchronous rounds of replication complicate flow cytometric definition of S phase parasites based upon DNA content. Furthermore, synchronization of blood-stage parasites, beyond age-range categories or morphological classification *via* microscopy, has been problematic because the most commonly used agents for chemically synchronising cells at a precise cell-cycle boundary are ineffective in *Plasmodium* [103].

Fortunately, in recent years significant technological advances have occurred that will facilitate the study of the *Plasmodium* cell cycle. The advent of bromodeoxyuridine (BrdU) labelling for *Plasmodium* [104] means that accurate detection of S phase entry and tracing of *de novo* DNA synthesis is now possible (Fig. 3c). CRK4 depletion in *P. falciparum* has been shown halt DNA replication [12] and might be exploited as a valuable synchronisation tool. Progress in reverse-genetic techniques such as Crispr/Cas9 [105], Selection linked integration (SLI) [106], and the DiCre system for conditional knockdown [107] have improved the genetic tractability and speed of genetic experiments in the parasite. Such techniques can undoubtedly help to further characterise the cyclin/CDK machinery and the elusive checkpoint systems in *Plasmodium*. Along with advances in imaging methods, such as improved deconvolution of widefield microscopy, super resolution techniques and spinning disk confocal microscopy, we are now better equipped than ever to study the cell cycles of *Plasmodium*.

## Conclusion

This review summarises our current knowledge about the events of the *Plasmodium* cell cycles at both the cellular and molecular levels, emphasising many areas of incomplete understanding. The cell cycles pursued by this parasite vary enormously at different life-cycle stages, and even the most accessible of these, which can be grown readily *in vitro* in human erythrocytes, urgently demands further study. This is especially so because of the great potential for novel drug targets in this area of *Plasmodium* biology. The increasing availability of experimental tools for such work means that there has never been a better time to advance our understanding of the cell cycle in this globally important human parasite.

## Abbreviations

APC: anaphase promoting complex
Ark: Aurora kinase
ARS: autonomously replicating sequence
BER: base excision repair
BrdU/CldU/IdU: bromo/chloro/iodo-deoxyuridine
CDK: cyclin dependent kinase
CDPK: calcium-dependent protein kinase
ChIP: chromatin immuno-precipitation
CP: centriolar plaque
CRK: CDK-related kinase
DSB: DNA double strand break
hpi: hours post-invasion
HR: homologous recombination
MCM: minichromosome maintenance protein
MMEJ: microhomology-mediated end joining
MMR: mismatch repair
Nek: NIMA kinase
NER: nucleotide excision repair
NHEJ: non-homologous end joining
ORC: Origin Recognition Complex
*Pb*: *Plasmodium berghei*
PCNA: proliferating cell nuclear antigen
*Pf*: *Plasmodium falciparum*
RFC: replication factor complex
SLI: Selection linked integration
